# Do patients with cN0 oral squamous cell carcinoma benefit from elective neck dissection? A large-scale population-based study

**DOI:** 10.1186/s12903-023-03632-5

**Published:** 2024-01-06

**Authors:** Qiuyu Wu, Yuanhang Xia, Ling Qiu, Shuqiong Wen, Qunxing Li, Xiang Gao, Wenrong Jiang, Tao Wang, Ping Ji, Zhanpeng Ou

**Affiliations:** 1 Chongqing Key Laboratory of Oral Diseases and Biomedical Sciences, Stomatological Hospital of Chongqing Medical University, No. 426 Songshi North Road, Yubei District, Chongqing, China; 2 Chongqing Municipal Key Laboratory of Oral Biomedical Engineering of Higher Education, Stomatological Hospital of Chongqing Medical University, Chongqing, China; 3 Department of Oral and Maxillofacial Surgery, Sun Yat-Sen Memorial Hospital, Guangzhou, China; 4 Department of Oral and Maxillofacial Surgery, Stomatological Hospital of Chongqing Medical University, Chongqing, China

**Keywords:** Oral squamous cell carcinoma, Clinical-nodal negative, Elective neck dissection, SEER database, Multiple subgroup analyses, Disease-specific survival, Overall survival

## Abstract

**Background:**

The neck management of clinical-nodal negative (cN0) oral squamous cell carcinoma (OSCC) remains controversial. Elective neck dissection (END) and observation are the main strategies, but it is still not clear who could benefit the most from END. The purpose of this study was to clarify the potential clinical factors that affect the therapeutic value of END and to explore the actual characteristics associated with benefit from END.

**Methods:**

Patients with cN0 OSCC were identified in the SEER database from 2000 to 2019. 5-year Overall survival (OS) and disease-specific survival (DSS) were analyzed using the Kaplan‒Meier method, and the hazard ratios (HRs) for survival were estimated using the Cox regression model. Multiple subgroup analyses of DSS and OS among different factors, comparing END and No END, were performed.

**Results:**

A total of 17,019 patients with cN0 OSCC were included. The basic survival analysis and Cox regression model showed that END increased the probability of 5-year DSS and OS and was an independent prognostic factor. However, among patients who underwent only primary tumor surgery, no significant differences were found between the END and No END groups in 5-year DSS (*P* = 0. 585) and OS (*P* = 0.465). Further subgroup analysis showed that primary sites and T stage, but not other factors, might influence the benefit of END. Significant differences were found for T1 (*P* < 0.001 for OS) and T2 (*P* = 0.001 for DSS and < 0.001 for OS) tongue squamous cell carcinoma (TSCC) but not for other primary tumor sites.

**Conclusion:**

This large-scale retrospective population-based cohort study suggests that not all patients with cN0 OSCC could benefit from END. Patients with cN0 TSCC are recommended to undergo END, especially with early-stage tumors.

**Supplementary Information:**

The online version contains supplementary material available at 10.1186/s12903-023-03632-5.

## Introduction

According to the GLOBOCAN 2020 database, there were 377,713 new cases (2.0% of the total) of lip and oral cavity cancer and 177,757 new deaths (1.8% of the total) from this condition in 2020, the majority of which were oral squamous cell carcinoma (OSCC) [1]. Surgical excision of the primary tumor is the major treatment for patients with OSCC according to the NCCN Clinical Practice Guidelines in Oncology for Head and Neck Cancers, combined with radiation therapy, chemotherapy and the newly introduced immunotherapy [2].

One of the defining features of OSCC is lymph node metastasis, which can be occult and dramatically affect the survival and prognosis of patients [3]. Although imaging technologies have evolved in decades, it is still impossible to detect all occult metastases in the cervical lymph nodes [4]. The best approach to neck management has not reached a consensus, especially for clinical-nodal negative (cN0) OSCC patients [5].

Elective neck dissection (END) and observation are the two main strategies for neck management, which are chosen mostly based on the judgments of surgeons [5]. Whether to perform END or just wait and observe has been debated for a long time but still remains controversial [6]. Many researchers found that END could significantly prolong the survival time and decrease regional recurrences in patients with tongue, floor of mouth, buccal mucosa or other sites of squamous cell carcinoma, advocating that END should be the upfront treatment to remove any occult metastasis [7, 8]. In contrast, other studies showed that the performance of END was not associated with improved rates of overall or disease-specific survival, especially in cT1-2 OSCC, and it was associated with higher rates of complications, including shoulder dysfunction, pain, and contour changes [9–11]. The conflict between these two decisions may be due to the lack of sufficient samples and multidimensional analysis of different kinds of patients with OSCC.

The Surveillance, Epidemiology, and End Results (SEER) database provides an appropriate opportunity to survey the necessity and benefit of END in patients with cN0 OSCC because of its large sample size and relatively comprehensive clinical records [12]. Based on the large-scale population of these patients, we thoroughly investigated the potential factors that modulate the benefit of END on patient survival and identified the optimal candidates who could gain maximum benefit from END with multiple subgroup analyses. To our knowledge, this retrospective study was conducted with the most updated data from the SEER database and the largest possible sample size. We sought to provide objective evidence for the management of the cN0 neck in patients with OSCC.

## Methods

### Data Sources

In this retrospective large-scale population-based study, the detailed information of patients from SEER database 17 registries (Nov 2021 Submission, 2000–2019) was downloaded using SEER*Stat 8.4.0 software with permission from NCI (reference number 12910-Nov2021). Patients who met the following criteria were extracted: (1) Histologic type ICD-O-3: 8050–8076, 8078, 8083, 8084, 8094; (2) primary sites: C02.0, C02.1, C02.2, C02.3, C02.8, C02.9, C03.0, C03.1, C03.9, C04.0, C04.9, C05.0, C06.0, C06.1, C06.2; (3) clinically node-negative (N0) neck. Exclusion criteria included (1) incomplete survival record; (2) incomplete END record; and (3) incomplete AJCC cancer staging record.

The variables investigated in this study included the age at diagnosis, year of diagnosis, sex, race recode, marital status at diagnosis, primary tumor site, derived AJCC T and M stages, SEER*Stat RX Summary-Surgery Primary Site, RX Summary-Scope Reg LN Sur, radiation recode, chemotherapy recode, survival months, vital status recode, cause of death to site recode and SEER cause-specific death classification. The primary study outcomes were overall survival (OS) and disease-specific survival (DSS), and the hazard ratios (HRs) was also calculated.This cohort study followed the Strengthening the Reporting of Observational Studies in Epidemiology (STROBE) reporting guideline.

### Statistical analysis

Clinical and demographic features were compared across subgroups using the chi-square or Fisher exact test. Overall survival (OS) time was calculated from diagnosis to death from any cause and was censored if the patient was alive at the last follow-up or up to 60 months. Disease-specific survival (DSS) time was calculated from diagnosis to death from “SEER cause-specific death classification”. Patients were censored if they died from other causes or were alive at the last follow-up or up to 60 months. Survival curves of OS and DSS were analyzed using the Kaplan‒Meier method, and survival differences between subgroups were compared using the log-rank test. Then, all significant factors were included in a multivariate analysis based on a multivariate Cox proportional hazards regression model that was used to estimate the HRs for survival. Statistical calculations were performed using IBM SPSS Statistics software (version 25.0, IBM Corp. US), and visualization was performed using the “survminer” and “forestmodel” packages in R (version 4.1.0). All statistical tests were 2-sided and considered significant at *P* < 0.05.

Since this study did not involve interactions with human subjects or the use of any personal identifying information, institutional review board approval for the use of this deidentified dataset was not needed.

## Results

### Baseline characteristics and survival analysis

A total of 17,019 patients with cN0 OSCC who met the inclusion criteria were identified. The median age at diagnosis was 67 years (range 6–85 +), and the mean follow-up time was 47.37 months. The majority of these patients were male (9448, 55.5%), white (14,540, 85.4%) and married (9059, 53.2%). END was performed in 4078 patients (24.0%), while the other 12,941 (76.0%) patients did not undergo END.

**Fig. 1:**
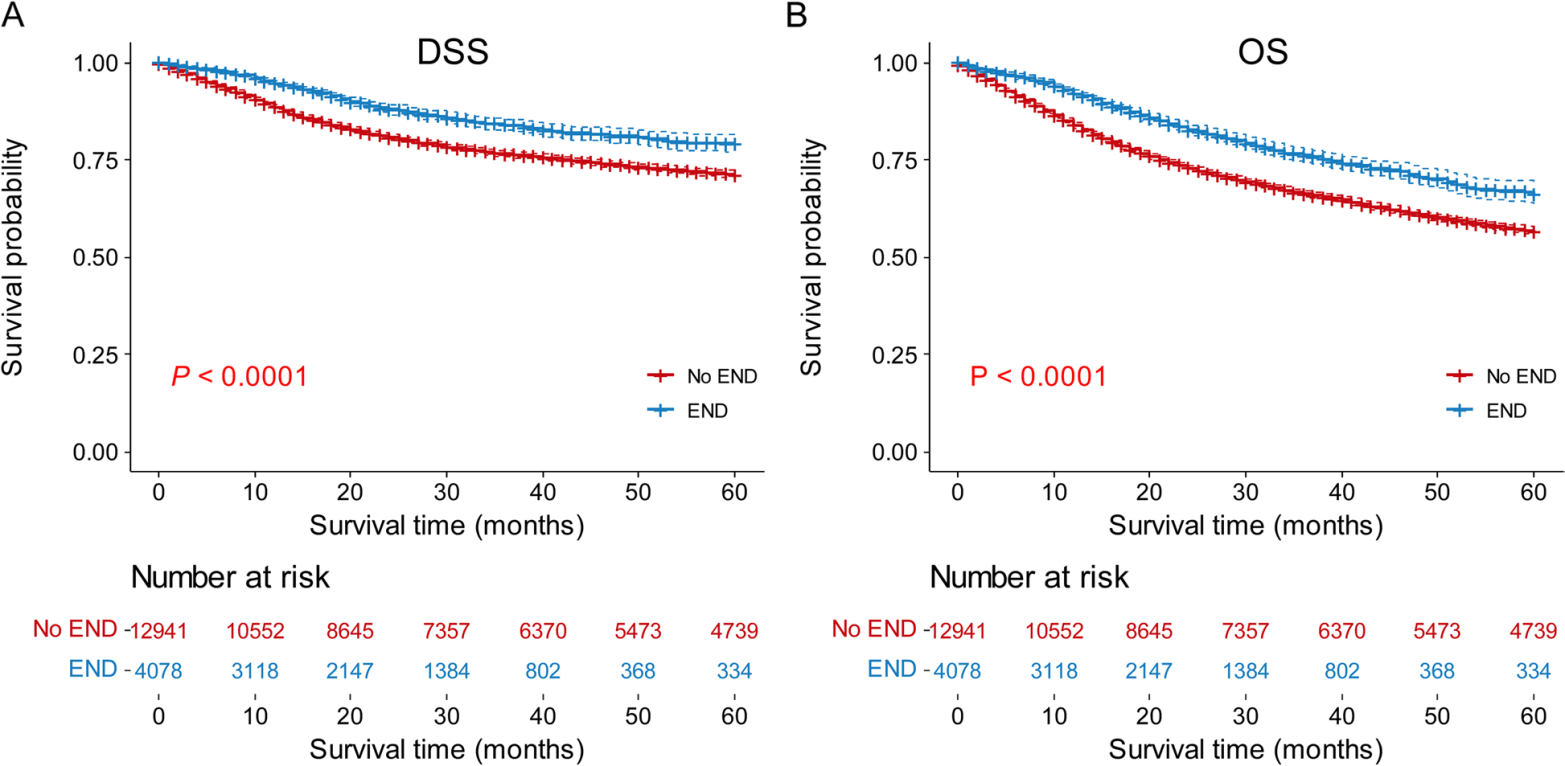
DSS (**A**) and OS (**B**) curves of patients with cN0 OSCC with or without END. Abbreviations: *DSS* Disease-specific survival, *END* Elective neck dissection, *OS* Overall survival, *OSCC* Oral squamous cell carcinoma

Significant differences in OS and DSS were found for END (Fig. 1), age, race, marital status, primary sites, T stage, M stage, surgery, radiation and chemotherapy (*P* < 0.001) but not for sex (*P* = 0.049 in DSS but = 0.74 in OS, Supplementary Figs. 1–2). The Cox regression model showed that age, race, marital status, primary sites, T stage, M stage, surgery, radiation, chemotherapy, and most importantly, END, were independent prognostic indicators (Supplementary Fig. 3). The END group was associated with prolonged DSS (HR, 0.72; 95% confidence interval [CI], 0.64–0.80; *P* < 0.001) and OS (HR, 0.77; 95% CI, 0.71–0.84; *P* < 0.001) compared with the No END group. As shown in Table 1, except for sex, the dead/alive ratio for age, race, marital status, primary sites, T stage, M stage, surgery, radiation, and chemotherapy were significantly different between the END group and the No END group, both for OS and DSS. These results were consistent with previous studies [13].

**Table 1:** Baseline characteristics of patients with cN0 OSCC

	Disease-Specific Survival								Overall Survival							
	END				No END				END				No END			
parameters	Alive	Dead	Total	*P*-value	Alive	Dead	Total	*P*-value	Alive	Dead	Total	*P*-value	Alive	Dead	Total	*P*-value
Sex								0.669^c^								0.669
				0.930^a^				0.096				0.123				0.488
Female	1524	190	1714(42^b^)		4454	1403	5857(45.3)		1434	280	1714(42)		3685	2172	5857(45.3)	
Male	2104	260	2364(58)		5475	1609	7084(54.7)		1934	430	2364(58)		4415	2669	7084(54.7)	
Age (y)								0.279								0.279
				** < 0.001**				**< 0.001**				**< 0.001**				**< 0.001**
≤ 50	522	47	569(14)		1061	166	1227(9.5)		510	59	569(14)		1004	223	1227(9.5)	
50–59	846	92	938(23)		1997	409	2406(18.6)		797	141	938(23)		1827	579	2406(18.6)	
60–69	1109	122	1231(30.2)		2711	618	3329(25.7)		1036	195	1231(30.2)		2361	968	3329(25.7)	
70–79	798	91	889(21.8)		2359	746	3105(24)		737	152	889(21.8)		1862	1243	3105(24)	
≥ 80	353	98	451(11.1)		1801	1073	2874(22.2)		288	163	451(11.1)		1046	1828	2874(22.2)	
Race								0.918								0.918
				**0.015**				**< 0.001**				**< 0.001**				**< 0.001**
White	2959	390	3349(82.1)		8616	2575	11,191(86.5)		2734	615	3349(82.1)		6988	4203	11,191(86.5)	
Black	161	22	183(4.5)		379	200	579(4.5)		152	31	183(4.5)		281	298	579(4.5)	
AI	23	2	25(0.6)		48	15	63(0.5)		18	7	25(0.6)		38	25	63(0.5)	
API	455	36	491(12)		786	219	1005(7.8)		434	57	491(12)		696	309	1005(7.8)	
Other	30	0	30(0.7)		100	3	103(0.8)		30	0	30(0.7)		97	6	103(0.8)	
Marital status								0.807								0.807
				**0.025**				**< 0.001**				**< 0.001**				**< 0.001**
Single	628	73	701(14.5)		1376	453	1829(17.1)		576	125	701(17.2)		1135	694	1829(14.1)	
Married	2019	228	2247(53.1)		5483	1329	6812(55.3)		1908	339	2247(55.1)		4680	2132	6812(52.6)	
Other	981	149	1130(32.4)		3070	1230	4300(27.6)		884	246	1130(27.7)		2285	2015	4300(33.2)	
T								**0.003**								**0.003**
				** < 0.001**				**< 0.001**				**< 0.001**				**< 0.001**
T1	1416	118	1534(37.6)		7128	1157	8285(64)		1333	201	1534(37.6)		6112	2173	8285(64)	
T2	1172	131	1303(32)		1885	886	2771(21.4)		1093	210	1303(32)		1399	1372	2771(21.4)	
T3	332	53	385(9.4)		319	290	609(4.7)		303	82	385(9.4)		197	412	609(4.7)	
T4	708	148	856(21)		597	679	1276(9.9)		639	217	856(21)		392	884	1276(9.9)	
M								0.605								0.605
				**0.003**				**< 0.001**				**< 0.001**				**< 0.001**
M0	3619	445	4064(99.7)		9879	2929	12,808(99)		3362	702	4064		8076	4732	12,808	
M1	7	5	12(0.3)		30	75	105(0.8)		5	7	12		12	93	105	
MX	2	0	2(0)		20	8	28(0.2)		1	1	2		12	16	28	
Primary sites								0.910								0.910
				**0.007**				**< 0.001**				**< 0.001**				**< 0.001**
Tongue	2088	216	2304(56.5)		5768	1465	7233(55.9)		1977	327	2304(56.5)		4905	2328	7233(55.9)	
Buccal mucosa	309	45	354(8.7)		834	322	1156(8.9)		283	71	354(8.7)		661	495	1156(8.9)	
Floor of mouth	501	71	572(14)		1407	422	1829(14.1)		442	130	572(14)		1046	783	1829(14.1)	
Gum	572	94	666(16.3)		1405	481	1886(14.6)		524	142	666(16.3)		1090	796	1886(14.6)	
Hard palate	66	9	75(1.8)		373	225	598(4.6)		57	18	75(1.8)		292	306	598(4.6)	
Others	92	15	107(2.6)		142	97	239(1.8)		85	22	107(2.6)		106	133	239(1.8)	
Surgery								**< 0.001**								**< 0.001**
				0.055				**< 0.001**				**0.007**				**< 0.001**
No	7	3	10(0.2)		982	1157	2139(16.5)		5	5	10(0.2)		534	1605	2139(16.5)	
Yes	3621	447	4068(99.8)		8947	1855	10,802(83.5)		3363	705	4068(99.8)		7566	3236	10,802(83.5)	
Radiation								**0.027**								**0.027**
				**0.014**				**< 0.001**				0.396				**< 0.001**
No	2373	268	2641(64.8)		8376	1873	10,249(79.2)		2191	450	2641(64.8)		6979	3270	10,249(79.2)	
Yes	1255	182	1437(35.2)		1553	1139	2692(20.8)		1177	260	1437(35.2)		1121	1571	2692(20.8)	
Chemotherapy								1.000								1.000
				** < 0.001**				**< 0.001**				**< 0.001**				**< 0.001**
No	3382	386	3768(92.4)		9394	2495	11,889(91.9)		3144	624	3768(92.4)		7730	4159	11,889(91.9)	
Yes	246	64	310(7.6)		535	517	1052(8.1)		224	86	310(7.6)		370	682	1052(8.1)	

*AI* American Indian/Alaska Native, *API* Asian or Pacific Islander, *B* Black, *END* Elective neck dissection, *TSCC* Tongue squamous cell carcinoma, *W* White

^a^Chi-square test comparing survival rates between variables within each parameter

^b^proportion (%) of each variable within each parameter

^c^Chi-square test comparing the proportion of each parameter between END and no END

### Subgroup survival analysis

**Fig. 2:**
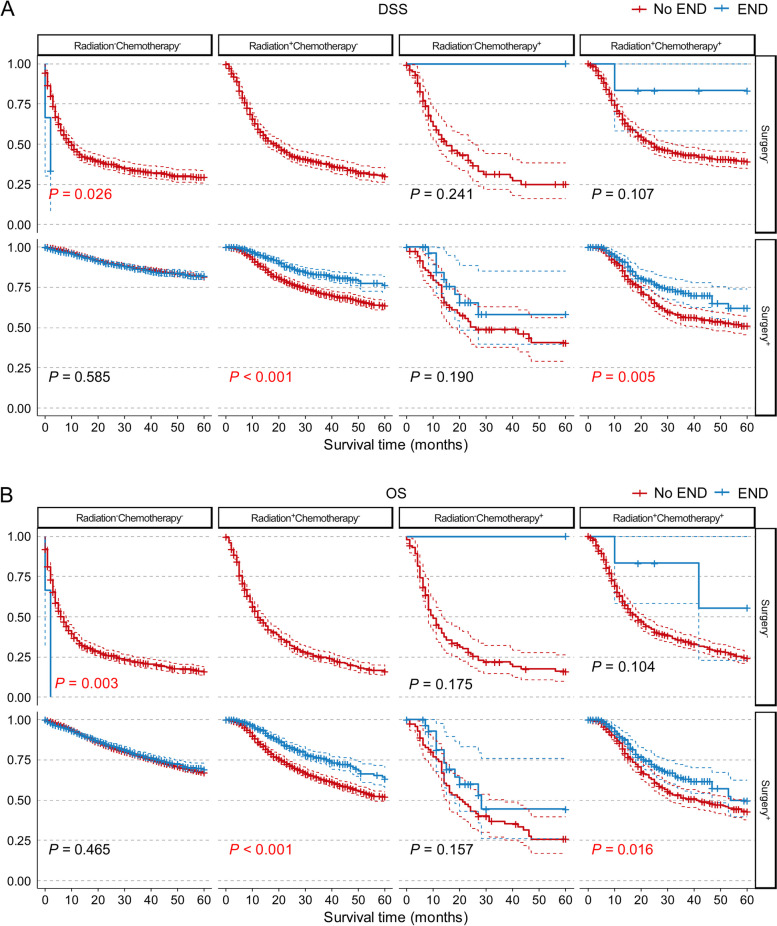
DSS (**A**) and OS (**B**) curves of patients with cN0 OSCC according to different treatment categories with END subgroup analysis. Abbreviations: *DSS* Disease-specific survival, *END* Elective neck dissection, *OS* Overall survival, *OSCC* Oral squamous cell carcinoma

From the results above, it seems that END could increase the 5-year survival rates in cN0 OSCC patients, but it is still not clear which kind of patients could benefit most from END or who could not benefit from END. To further identify the actual candidates, we performed a subgroup analysis of all of the independent prognostic factors. Indeed, END significantly increased the probability of 5-year DSS and OS in most subgroups of age, race, marital status, primary sites, T stage, M stage, surgery, radiation and chemotherapy (*P* < 0.001 for both DSS and OS), except for age < 50 (*P* = 0.214 for DSS and *P* = 0.074 for OS), age 50–80 (*P* = 0.086 for DSS and *P* = 0.505 for OS), American Indian/Alaska Native (AI, *P* = 0.320 for DSS and *P* = 0.979 for OS) and unknown race (*P* = 0.382 for DSS and *P* = 0.250 for OS) and unknown M stage (MX, *P* = 0.442 for DSS and *P* = 0.883 for OS, Fig. 2, Supplementary Figs. 4–5).

To our surprise, the results of the surgery and END subgroup analyses showed that there was no difference in DSS and OS among the patients who underwent surgery, regardless of whether they received END (Supplementary Fig. 6). In contrast, patients had better survival if they underwent surgery in both the END and No END groups (Supplementary Fig. 6). In addition, the constituent ratios between the END and No END groups displayed significant differences only for T stage and surgery. A total of 99.8% of patients in the END group underwent surgery, while only 83.5% of patients did in the No END group (*P* < 0.001) (Table 1). These results indicated that the benefit brought by END might mostly come from undergoing surgery and further confirmed that not all patients with cN0 OSCC could benefit from END.

### Further exploration of potential subgroups that benefited from END

To identify the patients who received the most benefit from END, we first performed subgroup analysis of different treatment strategies. As shown in Supplementary Fig. 7, there was a dramatic difference among these strategies, with surgery only being the optimal one for both DSS and OS. Among these subgroups, END outweighed No END only in the Surgery^+^Radiation^+^Chemotherapy^−^ group (*P* < 0.001 for both DSS and OS) and Surgery^+^Radiation^+^Chemotherapy^+^ group (*P* = 0.005 for DSS and *P* = 0.026 for OS). Interestingly, in the only surgery group, END still showed little difference from No END both in DSS (*P* = 0.585) and OS (*P* = 0.465), which further proved our assumption (Fig. 2).

**Fig. 3:**
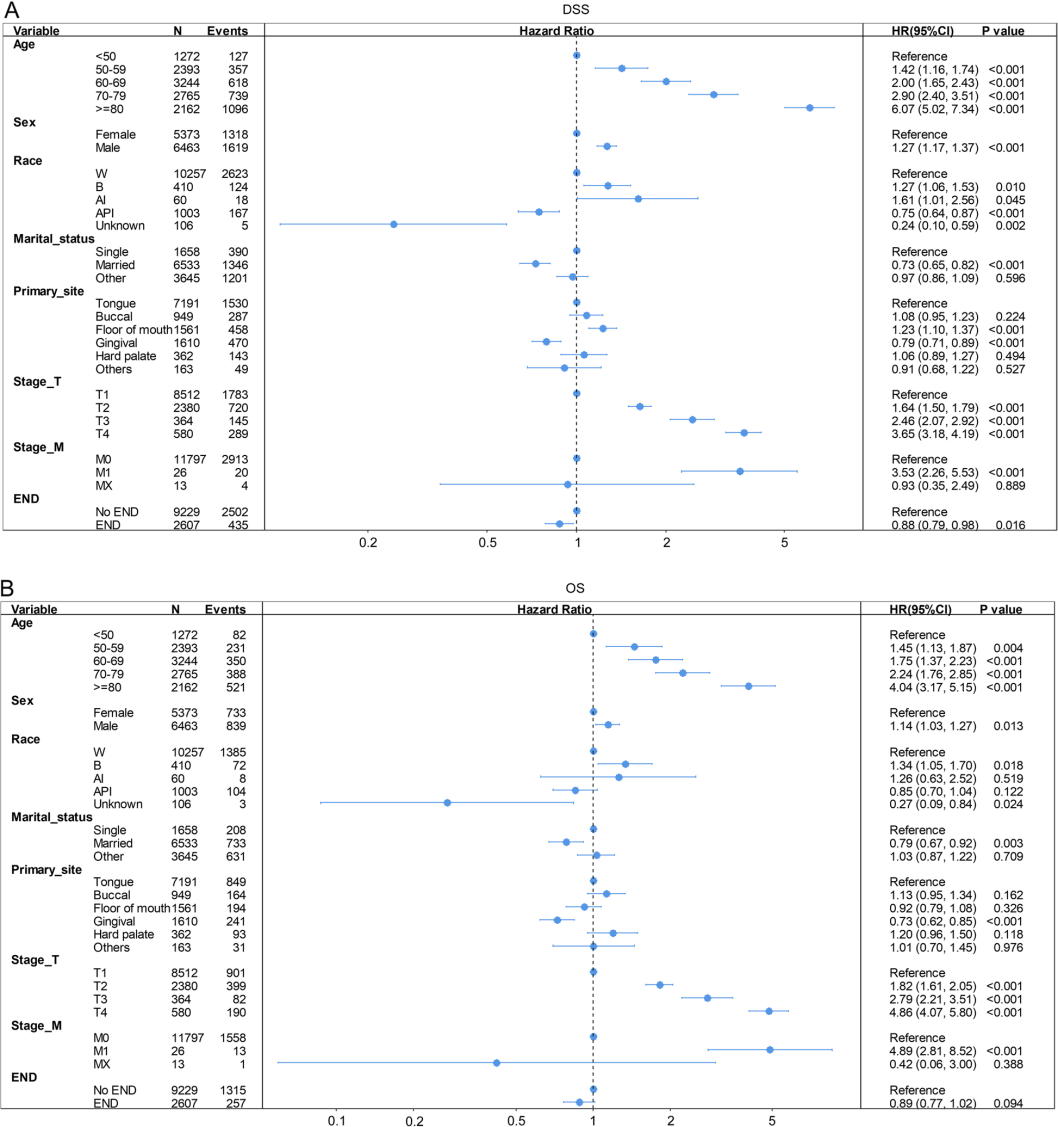
Forest plots summarizing hazard ratios for DSS (**A**) and OS (**B**) of patients who only underwent primary site surgery without radiation and chemotherapy. Abbreviations: *AI* American Indian/Alaska Native, *API* Asian or Pacific Islander, *B* black, *CI* Confidence interval, *DSS* Disease-specific survival, *END* Elective neck dissection, *HR* Hazard ratio, *MX* Unknown M stage, *OS* Overall survival

Since radiation and chemotherapy could greatly interfere with the survival analysis of END, we excluded these patients and focused on the patients who merely underwent primary site surgery. A total of 11,836 patients were identified, 45.4% of whom were female and 54.6% of whom were male. Their median age was 66 years (range 6–85 +), and the majority were also white (10,257, 86.7%) and married (6533, 55.2%). There were a total of 9229 (78.0%) patients in the END group and 2607 (22.0%) in the No END group. We estimated the potential factors using a Cox regression model in these separate groups of patients. As displayed in Fig. 3, variables including age, sex, race, marital status, primary site, T stage, M stage and END were independent prognostic factors.

**Fig. 4:**
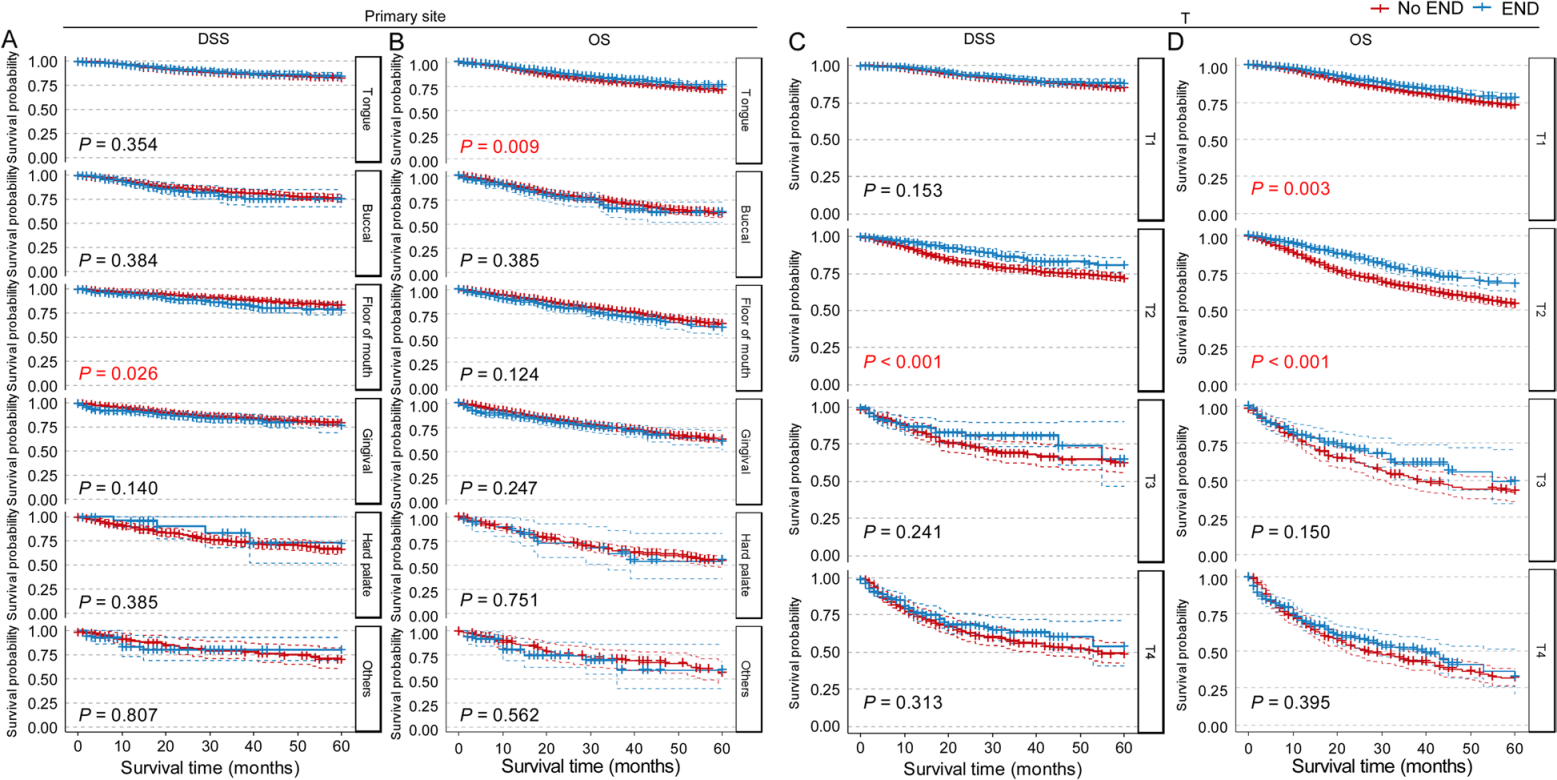
Subgroup survival analysis of END in cN0 OSCC patients who only underwent primary site surgery without radiation and chemotherapy according to the primary site (**A**, **B**) or T stage (**C**,** D**). Abbreviations: *DSS* Disease-specific survival, *END* Elective neck dissection, *OS* Overall survival, *OSCC* Oral squamous cell carcinoma

**Fig. 5:**
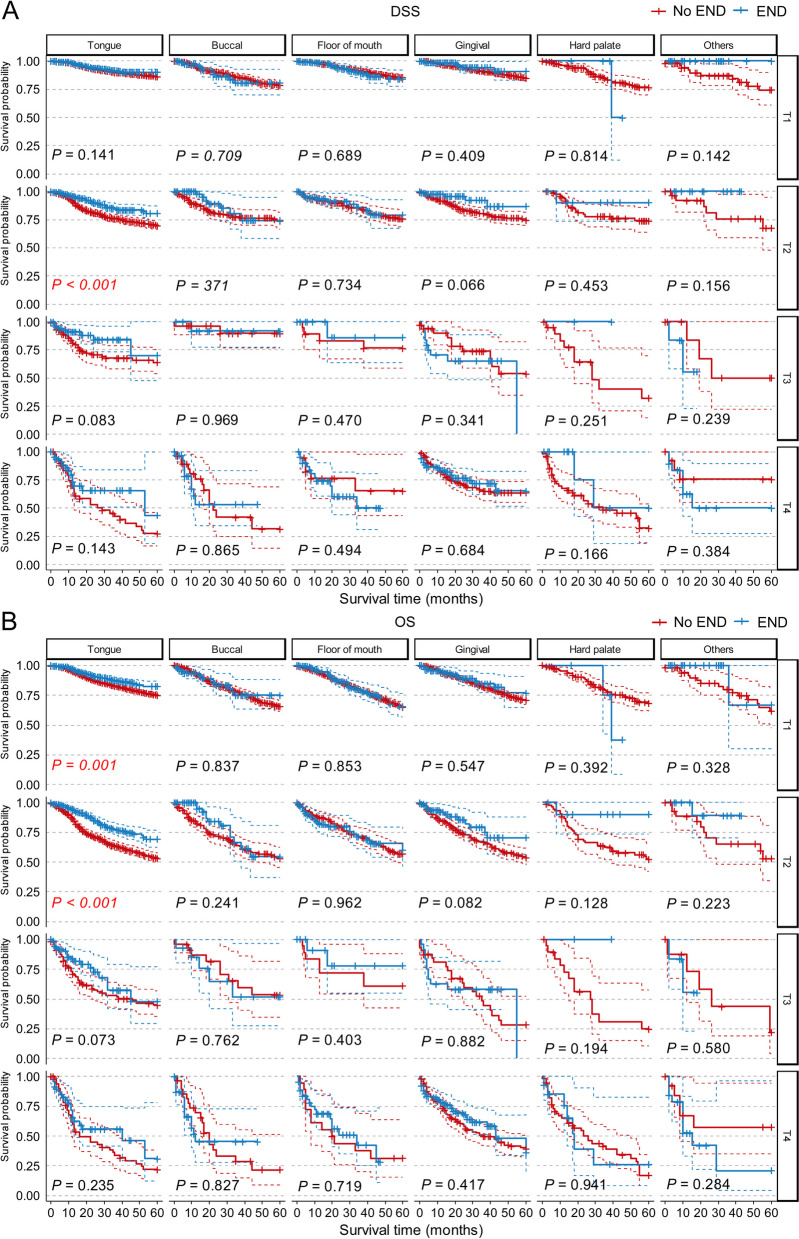
DSS (**A**) and OS (**B**) curves represent the cross-subgroup survival analysis of END in cN0 OSCC patients who only underwent primary site surgery without radiation and chemotherapy according to the primary sites and T stages. Abbreviations: *DSS* Disease-specific survival, *END* Elective neck dissection, *OS* Overall survival, *OSCC* Oral squamous cell carcinoma

We then performed subgroup analysis of DSS and OS for these factors and surprisingly found that for most indicators, the difference in DSS and OS between the END group and the No END group was no longer significant, including sex, age, race, marital status and M stage (Supplementary Figs. 8–9). However, for primary site and T stage, END showed better results for tongue (*P* = 0.009 for OS), T1 (*P* = 0.003 for OS) and T2 (*P* < 0.001 for both DSS and OS, Fig. 4). Therefore, we focused on these two independent indicators and conducted cross-subgroup survival analysis in multiple subgroups, as shown in Fig. 5. The results showed that in tongue squamous cell carcinoma (TSCC), END lead to better prognostic outcomes in T1 (*P* = 0.001 for OS) and T2 (*P* < 0.001 for both DSS and OS). In cN0 T3 and T4 TSCC patients, the DSS and OS curves also showed a trend toward a benefit from END, but the differences were not significant, perhaps because of the lack of samples (T3: *n* = 197, 2.7%; T4: *n* = 99, 1.3%). There was no significant difference in DSS or OS between the END and No END groups at other primary sites. The detailed characteristics of the TSCC patients are listed in Table 2. All of these results suggested that patients with TSCC may benefit from END, espesially with early-stage tumors.

**Table 2:** Baseline characteristics of patients with cN0 TSCC only performed primary sites surgery without radiation and chemotherapy

	Disease-Specific Survival								Overall Survival							
	END				no END				END				no END			
parameters	Alive	Dead	Total	*P*-value	Alive	Dead	Total	*P*-value	Alive	Dead	Total	*P*-value	Alive	Dead	Total	*P*-value
Sex								0.670^c^								0.670
				0.933^a^				0.077				0.438				0.101
Female	646	56	702(43.8^b^)		2294	314	2608(46.7)		617	85	702(43.8)		2016	592	2608(46.7)	
Male	829	73	902(56.2)		2573	406	2979(53.3)		781	121	902(56.2)		2247	732	2979(53.3)	
Age (y)								0.583								0.583
				**0.005**				**< 0.001**				**< 0.001**				**< 0.001**
≤ 50	263	12	275(17.1)		682	44	726(13)		259	16	275(17.1)		658	68	726(13)	
50–59	373	26	399(24.9)		1085	120	1205(21.6)		360	39	399(24.9)		1033	172	1205(21.6)	
60–69	434	42	476(29.7)		1386	167	1553(27.8)		413	63	476(29.7)		1268	285	1553(27.8)	
70–79	279	28	307(19.1)		1030	168	1198(21.4)		265	42	307(19.1)		864	334	1198(21.4)	
≥ 80	126	21	147(9.2)		684	221	905(16.2)		101	46	147(9.2)		440	465	905(16.2)	
Race								0.954								0.954
				**0.046**				**0.016**				**0.044**				**< 0.001**
White	1238	115	1353(84.4)		4223	637	4860(87)		1165	188	1353(84.4)		3673	1187	4860(87)	
Black	36	3	39(2.4)		125	26	151(2.7)		36	3	39(2.4)		102	49	151(2.7)	
AI	6	0	6(0.4)		19	3	22(0.4)		5	1	6(0.4)		16	6	22(0.4)	
API	179	11	190(11.8)		435	53	488(8.7)		176	14	190(11.8)		408	80	488(8.7)	
Other	16	0	16(1)		65	1	66(1.2)		16	0	16(1)		64	2	66(1.2)	
Marital status								0.677								0.677
				0.775				**< 0.001**				0.944				**< 0.001**
Single	253	21	274(17.1)		692	96	788(14.1)		240	34	274(17.1)		618	170	788(14.1)	
Married	845	78	923(57.5)		2810	336	3146(56.3)		805	118	923(57.5)		2533	613	3146(56.3)	
Other	377	30	407(25.4)		1365	288	1653(29.6)		353	54	407(25.4)		1112	541	1653(29.6)	
T								**0.001**								**0.001**
				**< 0.001**				**< 0.001**				**< 0.001**				**< 0.001**
T1	845	54	899(56)		4150	489	4639(83)		813	86	899(56)		3694	945	4639(83)	
T2	516	52	568(35.4)		618	171	789(14.1)		487	81	568(35.4)		498	291	789(14.1)	
T3	80	10	90(5.6)		77	30	107(1.9)		70	20	90(5.6)		56	51	107(1.9)	
T4	34	13	47(2.9)		22	30	52(0.9)		28	19	47(2.9)		15	37	52(0.9)	
M								1.000								1.000
				**< 0.001**				**0.001**				**< 0.001**				**< 0.001**
M0	1474	127	1601(99.8)		4852	713	5565(99.6)		1398	203	1601(99.8)		4253	1312	5565(99.6)	
M1	1	2	3(0.2)		6	6	12(0.2)		0	3	3(0.2)		2	10	12(0.2)	
MX	0	0	0(0)		9	1	10(0.2)		0	0	0(0)		8	2	10(0.2)	

*AI* American Indian/Alaska Native, *API* Asian or Pacific Islander, *B* Black, *END* Elective neck dissection, *TSCC* Tongue squamous cell carcinoma, *W* White

^a^Chi-square test comparing survival rates between variables within each parameter

^b^proportion (%) of each variable within each parameter

^c^Chi-square test comparing the proportion of each parameter between END and no END

## Discussion

Based on the large-population survival analysis of patients with cN0 OSCC acquired from the SEER database, we found that END was an independent prognostic factor. Surprisingly, in the subgroup analysis, END did not affect survival in patients who underwent primary site surgery. After excluding the influence of radiation or chemotherapy on survival, we found that not all patients with cN0 OSCC could definitely benefit from END. Further analysis showed that patients with TSCC, even those with early-stage tumors, could benefit from END, but END did not lead to significantly better survival results for tumors arising from other sites.

In recent decades, researchers around the world have continuously focused on END in OSCC patients, especially those with early-stage tumors. Before 2010, four randomized controlled trials (RCTs) were carried out with small samples (only 67–71), three of which did not find differences in survival between END or observation [14–17]. A surgical team from Brazil found that patients treated with elective supraomohyoid neck dissection had a significant benefit in terms of disease-free survival (DFS) only in those whose tumor thickness was > 4 mm and a later stage, leaving the question unanswered [16]. In 2015, D’Cruz AK and his team performed the largest single-center RCT, the Mumbai trial [18], comparing END and therapeutic node dissection (watchful waiting followed by neck dissection for nodal relapse). Their results showed an improved rate of overall survival (80.0% vs. 67.5%, *P* = 0.01) and disease-free survival with END relative to those in the therapeutic surgery group (69.5% vs. 45.9%, *P* < 0.001), with similar complication rates (6.6% vs. 3.6%). Nevertheless, these studies could not provide conclusive evidence because of the low quality of the trials [19]. Later, the high-quality SEND study [19] in 2019 with 27 hospitals in the UK and 250 randomized patients indicated that those who underwent END had a lower risk of death or local recurrence, even with small tumors. However, the lack of enough samples from extensive areas still limits the generalization of END in cN0 OSCC patients.

Many researchers have conducted meta-analyses to summarize the effect of END from a larger cohort [20, 21], but the inherent heterogeneity among different studies, especially between RCTs and retrospective studies, makes it difficult to provide convincing conclusions [22]. Three meta-analyses, which included only the RCTs mentioned above, were conducted in 2011 [23], 2015 [24] and 2019 [25]. Their results revealed that END significantly reduced the risk of regional recurrences and the risk of disease-specific death and revealed a longer OS and DFS compared to observation. These studies, however, included low-quality RCTs, leading to their inferior reliability.

The SEER Program of the National Cancer Institute is a relatively comprehensive source of information on cancer incidence and survival in the United States, covering 48% of the U.S. population currently [25]. Several studies have investigated cN0 OSCC patients within this database [26]. Alimujiang et al. performed two SEER-based analyses comparing END and observation in cT1N0 or T2N0 OSCC patients separately [13, 27]. Through survival analysis and the Cox regression model, they showed that END was an independent prognostic indicator, improving both DSS and OS. These results are in accordance with our findings, but in our study, we further explored the potential factors that affect the survival of END-treated patients and the actual characteristics that predict a benefit from END.

To our knowledge, our study is the first to evaluate the effect of END in cN0 OSCC with multidimensional subgroup analysis. However, there are still several shortcomings of this study. An analysis of pathological grade was not included because the records of pathological grade were missing in most cases, and those with this record were censored at 2 years. Another disadvantage lies in the missing data of the actual affected lymph node distribution in different areas of the neck in those who underwent END, making it impossible to elucidate which district should be surgically excised among the different sites [28].

## Conclusions

To conclude, primary tumor site and T stage are the essential factors that influence the benefit of END on patient survival, and END is recommended in patients with cN0 TSCC, especially those with early-stage tumors. In the future, the potential immunological or molecular mechanism of END should be clarified, and large-scale multicenter RCTs investigating new surgical methods, such as sentinel lymph node dissection (SLND), are urgently needed [29].

### Supplementary Information

**Additional file 1: Supplementary Figure 1.** DSS curves of patients with cN0 OSCC according to (A) sex, (B) age group, (C) race, (D) marital status, (E) T, (F) M, (G) primary sites, (H) surgery, (I) radiation, (J) chemotherapy.** Supplementary Figure 2.** OS curves of patients with cN0 OSCC according to (A) sex, (B) age group, (C) race, (D) marital status, (E) T, (F) M, (G) primary sites, (H) surgery, (I) radiation, (J) chemotherapy. **Supplementary Figure 3.** Forest plots summarizing HR for (A) DSS and (B) OS.** Supplementary Figure 4. **DSS curves of patients with cN0 OSCC according to (A) age, (B) race, (C) primary sites, (D) T, (D) M, (E) Radiation, (F) Chemotherapy with END subgroups analysis. **Supplementary Figure 5. **OS curves of patients with cN0 OSCC according to (A) age, (B) race, (C) primary sites, (D) T, (D) M, (E) Radiation, (F) Chemotherapy with END subgroups analysis. **Supplementary Figure 6. **Subgroup analysis of END and surgery of patients with cN0 OSCC. **Supplementary Figure 7. **DSS and OS curves of patients with cN0 OSCC according to different treatment categories.** Supplementary Figure 8. **DSS of cN0 OSCC patients only performed primary sites surgery without radiation and chemotherapy according to (A) primary sites, (B) age, (C) race, (D) marital status and (E) M with END subgroups analysis. **Supplementary Figure 9.** OS of cN0 OSCC patients only performed primary sites surgery without radiation and chemotherapy according to (A) primary sites, (B) age, (C) race, (D) marital status and (E) M with END subgroups analysis.

## Data Availability

All data used in this paper (individual case listings as well as US population mortality data) may be accessed and analysed via the SEER*Stat web program following the submission of a request for access to the data at https://seer.cancer.gov/seertrack/data/request/. Further information is available from the corresponding author upon request.

## Abbreviations

AI: American Indian/Alaska Native
API: Asian or Pacific Islander
B: Black
CI: Confidence interval
cN0: Clinical-nodal negative
DSS: Disease-specific survival
END: Elective neck dissection
HRs: Hazard ratios
MX: Unknown M stage
OS: Overall survival
OSCC: Oral squamous cell carcinoma
RCTs: Randomized controlled trials
SEER: The Surveillance, Epidemiology, and End Results
SLND: Sentinel lymph node dissection
TSCC: Tongue squamous cell carcinoma
W: White
