# Supplementary material for: Do patients with cN0 oral squamous cell carcinoma benefit from elective neck dissection? A large-scale population-based study
Source: BMC Oral Health. 2024 Jan 6;24:32. doi: 10.1186/s12903-023-03632-5 (PMC10771637; doi:10.1186/s12903-023-03632-5)
Supplement: Supplementary file 1 — Additional file 1: Supplementary Figure 1. DSS curves of patients with cN0 OSCC according to (A) sex, (B) age group, (C) race, (D) marital status, (E) T, (F) M, (G) primary sites, (H) surgery, (I) radiation, (J) chemotherapy. Supplementary Figure 2. OS curves of patients with cN0 OSCC according to (A) sex, (B) age group, (C) race, (D) marital status, (E) T, (F) M, (G) primary sites, (H) surgery, (I) radiation, (J) chemotherapy. Supplementary Figure 3. Forest plots summarizing HR for (A) DSS and (B) OS. Supplementary Figure 4. DSS curves of patients with cN0 OSCC according to (A) age, (B) race, (C) primary sites, (D) T, (D) M, (E) Radiation, (F) Chemotherapy with END subgroups analysis. Supplementary Figure 5. OS curves of patients with cN0 OSCC according to (A) age, (B) race, (C) primary sites, (D) T, (D) M, (E) Radiation, (F) Chemotherapy with END subgroups analysis. Supplementary Figure 6. Subgroup analysis of END and surgery of patients with cN0 OSCC. Supplementary Figure 7. DSS and OS curves of patients with cN0 OSCC according to different treatment categories. Supplementary Figure 8. DSS of cN0 OSCC patients only performed primary sites surgery without radiation and chemotherapy according to (A) primary sites, (B) age, (C) race, (D) marital status and (E) M with END subgroups analysis. Supplementary Figure 9. OS of cN0 OSCC patients only performed primary sites surgery without radiation and chemotherapy according to (A) primary sites, (B) age, (C) race, (D) marital status and (E) M with END subgroups analysis. [file 12903_2023_3632_MOESM1_ESM.docx]

**Supplementary Information**

Supplementary Figure 1: DSS curves of patients with cN0 OSCC according to (A) sex, (B) age group, (C) race, (D) marital status, (E) T, (F) M, (G) primary sites, (H) surgery, (I) radiation, (J) chemotherapy.

Supplementary Figure 2: OS curves of patients with cN0 OSCC according to (A) sex, (B) age group, (C) race, (D) marital status, (E) T, (F) M, (G) primary sites, (H) surgery, (I) radiation, (J) chemotherapy.

Supplementary Figure 3. Forest plots summarizing HR for (A) DSS and (B) OS.

Supplementary Figure 4: DSS curves of patients with cN0 OSCC according to (A) age, (B) race, (C) primary sites, (D) T, (D) M, (E) Radiation, (F) Chemotherapy with END subgroups analysis.

Supplementary Figure 5: OS curves of patients with cN0 OSCC according to (A) age, (B) race, (C) primary sites, (D) T, (D) M, (E) Radiation, (F) Chemotherapy with END subgroups analysis.

Supplementary Figure 6: Subgroup analysis of END and surgery of patients with cN0 OSCC.

Supplementary Figure 7: DSS and OS curves of patients with cN0 OSCC according to different treatment categories.

Supplementary Figure 8: DSS of cN0 OSCC patients only performed primary sites surgery without radiation and chemotherapy according to (A) primary sites, (B) age, (C) race, (D) marital status and (E) M with END subgroups analysis.

Supplementary Figure 9: OS of cN0 OSCC patients only performed primary sites surgery without radiation and chemotherapy according to (A) primary sites, (B) age, (C) race, (D) marital status and (E) M with END subgroups analysis.


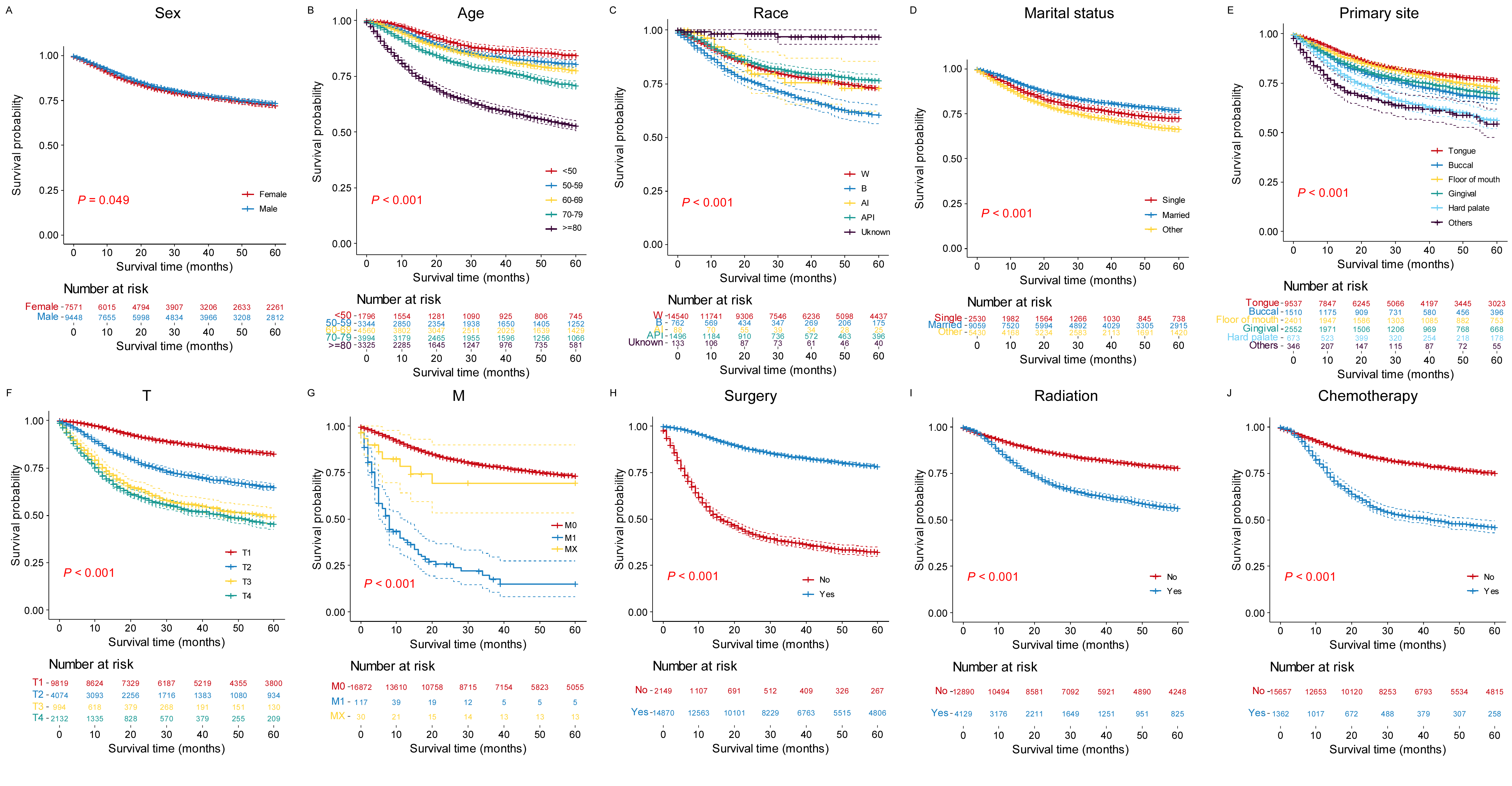


Supplementary Figure 1: DSS curves of patients with cN0 OSCC according to (A) sex, (B) age group, (C) race, (D) marital status, (E) T, (F) M, (G) primary sites, (H) surgery, (I) radiation, (J) chemotherapy.


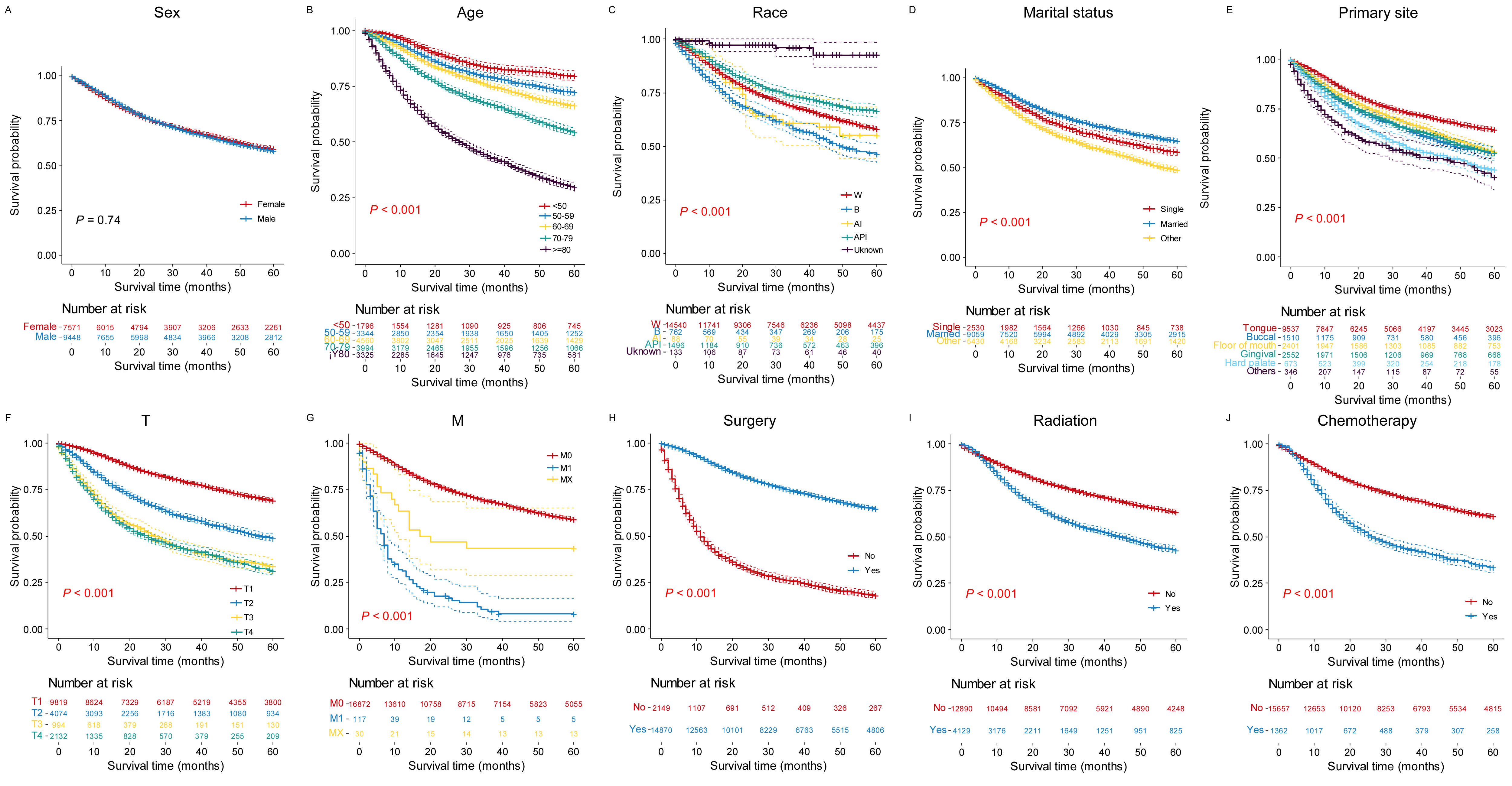


Supplementary Figure 2: OS curves of patients with cN0 OSCC according to (A) sex, (B) age group, (C) race, (D) marital status, (E) T, (F) M, (G) primary sites, (H) surgery, (I) radiation, (J) chemotherapy.


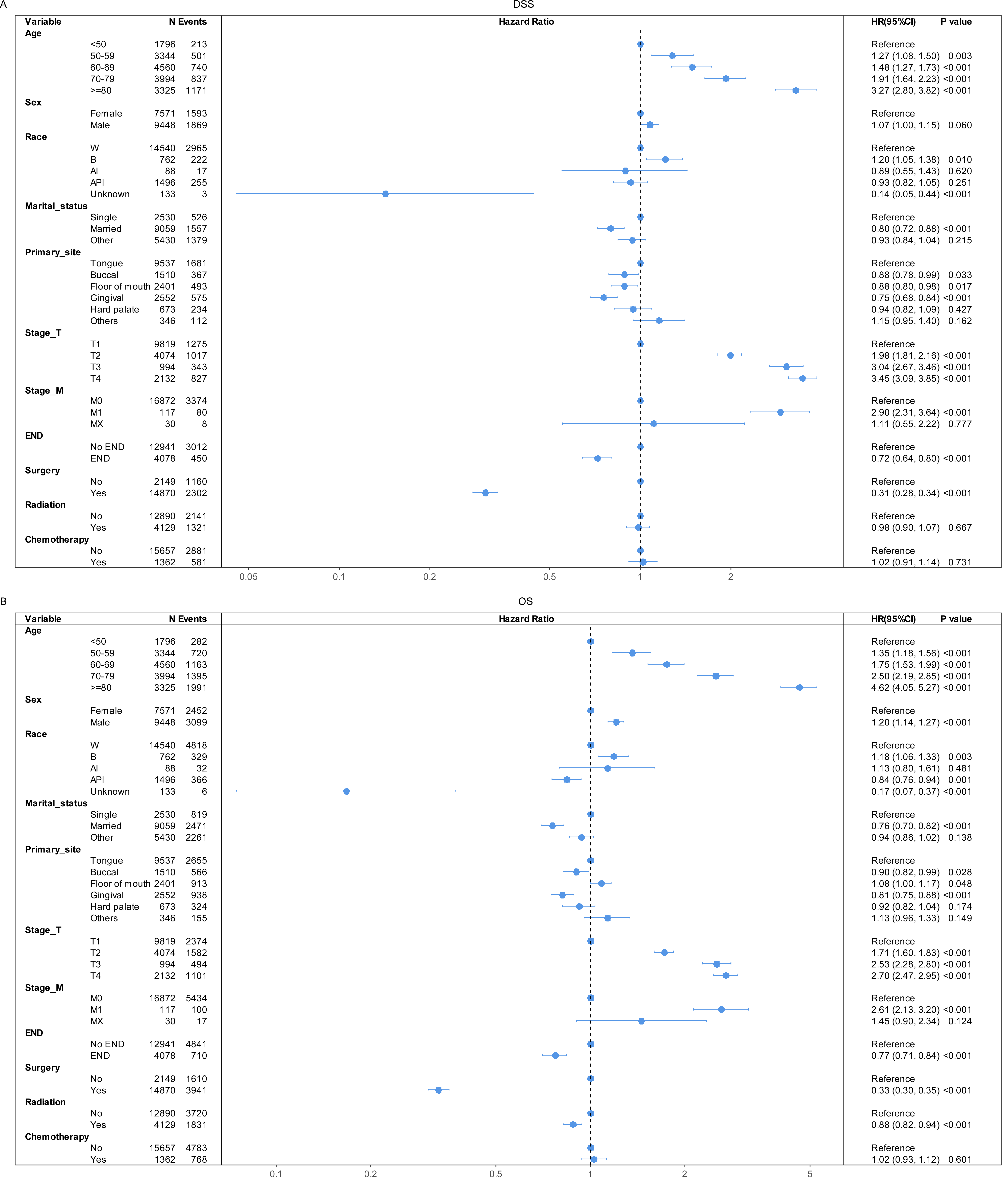


Supplementary Figure 3. Forest plots summarizing HR for (A) DSS and (B) OS.


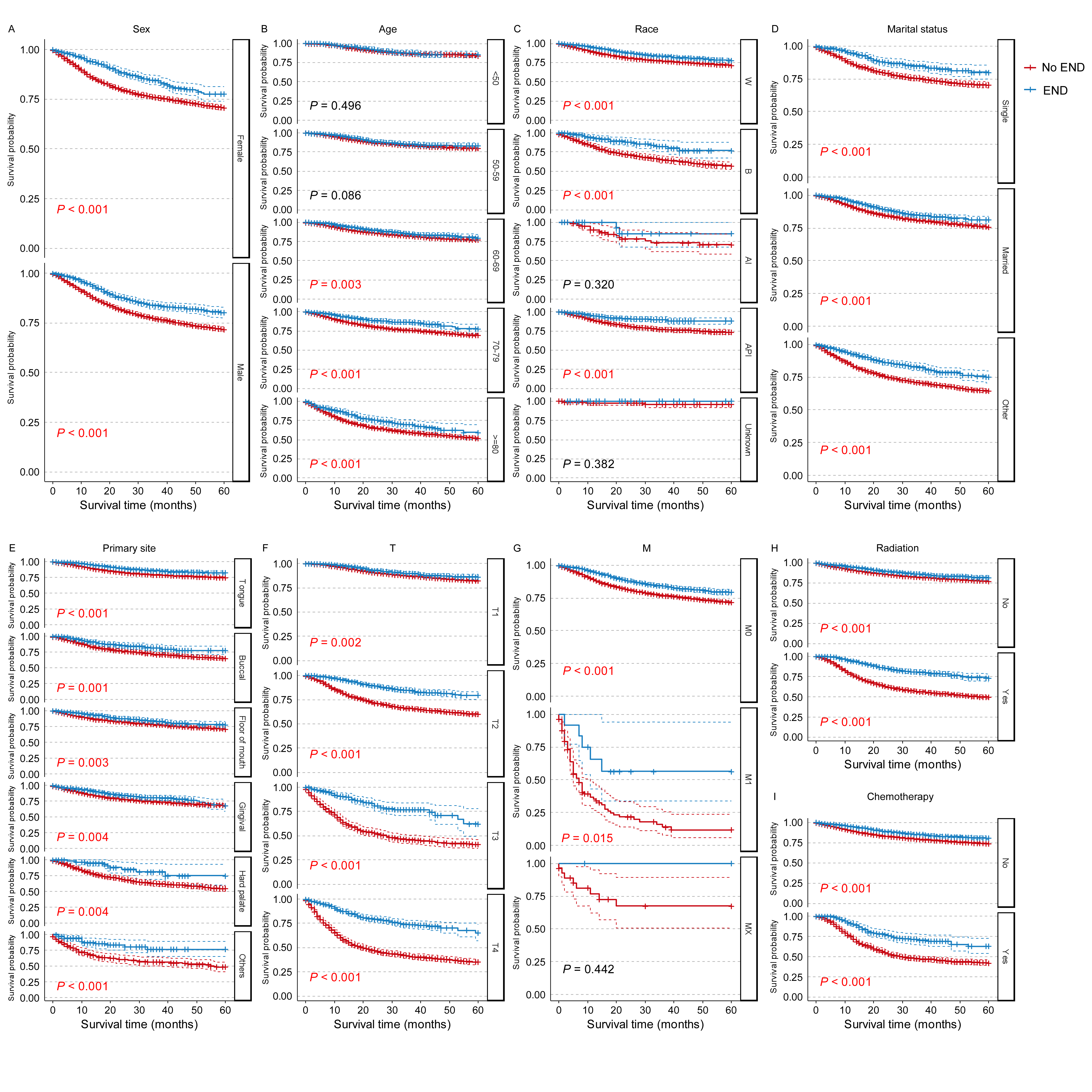


Supplementary Figure 4: DSS curves of patients with cN0 OSCC according to (A) age, (B) race, (C) primary sites, (D) T, (D) M, (E) Radiation, (F) Chemotherapy with END subgroups analysis.


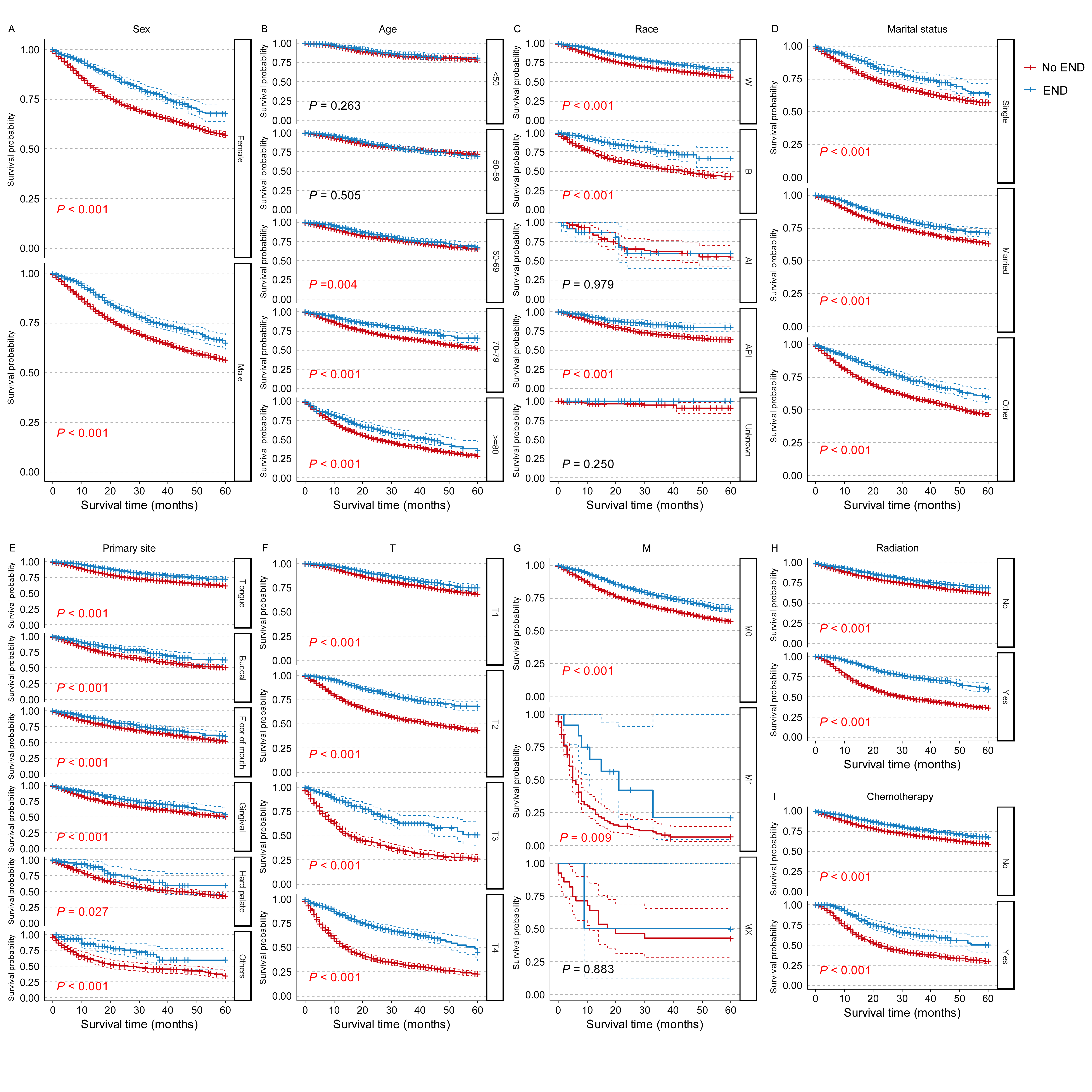


Supplementary Figure 5: OS curves of patients with cN0 OSCC according to (A) age, (B) race, (C) primary sites, (D) T, (D) M, (E) Radiation, (F) Chemotherapy with END subgroups analysis.


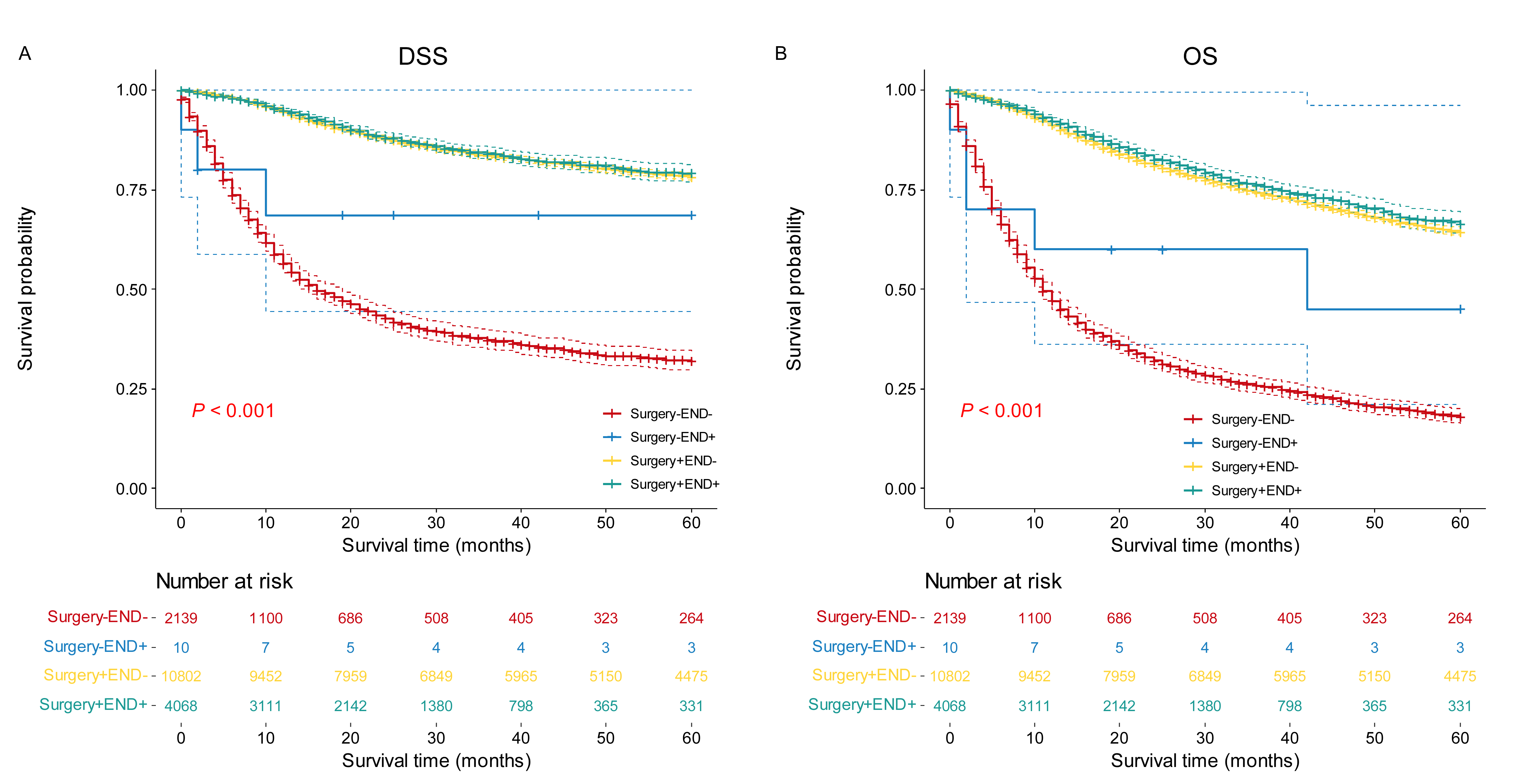


Supplementary Figure 6: Subgroup analysis of END and surgery of patients with cN0 OSCC.


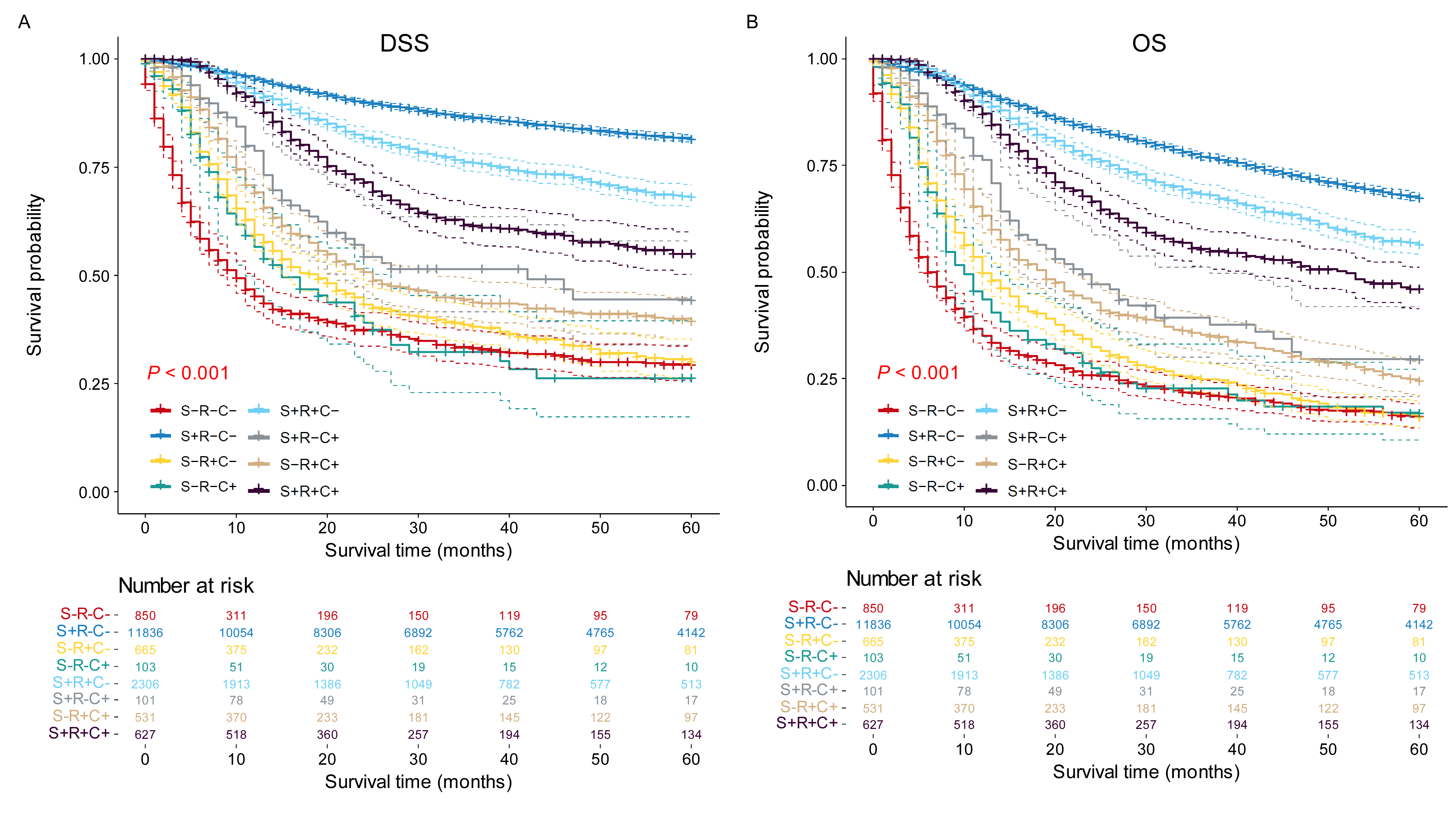


Supplementary Figure 7: DSS and OS curves of patients with cN0 OSCC according to different treatment categories.


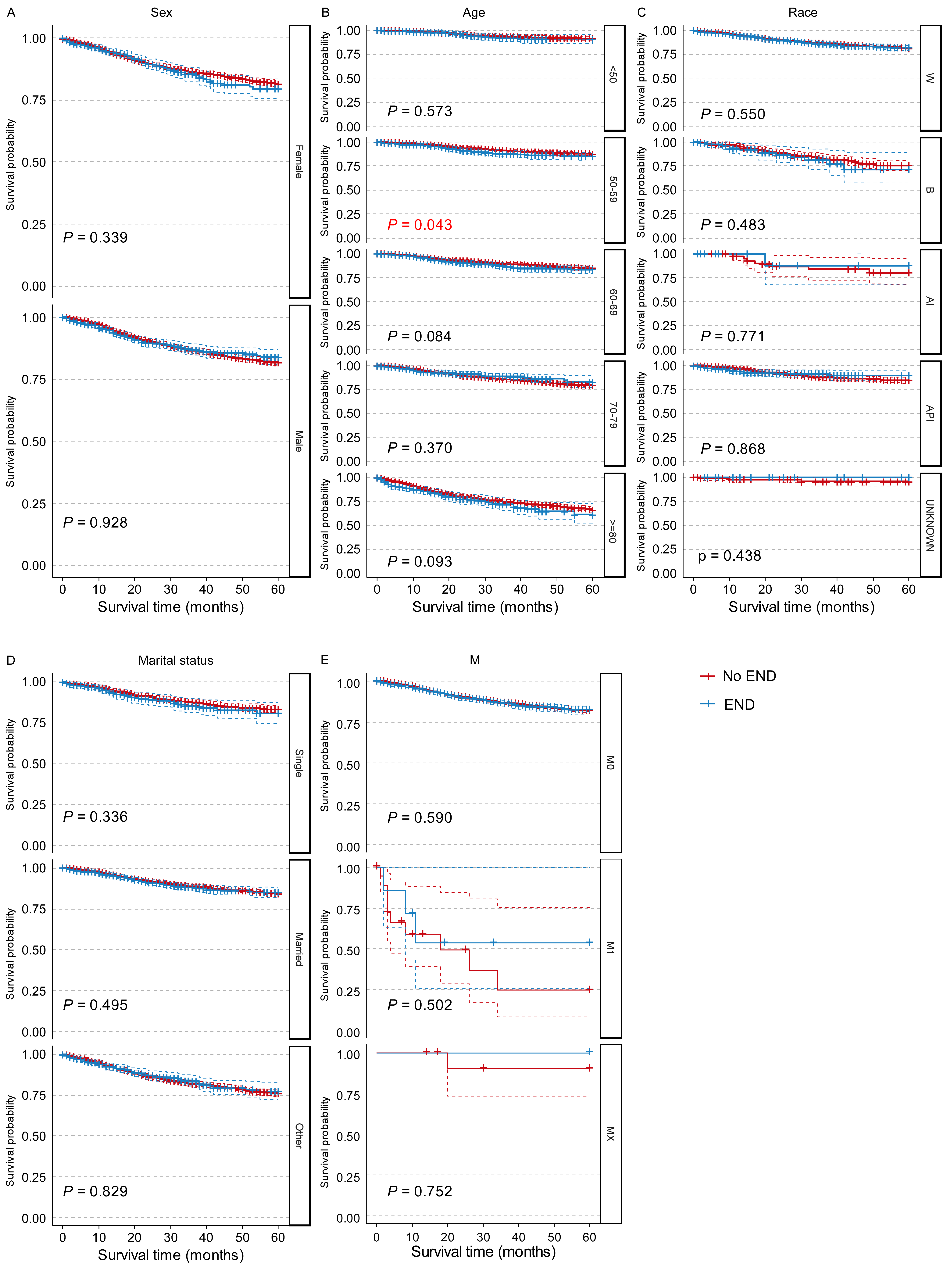


Supplementary Figure 8: DSS of cN0 OSCC patients only performed primary sites surgery without radiation and chemotherapy according to (A) primary sites, (B) age, (C) race, (D) marital status and (E) M with END subgroups analysis.


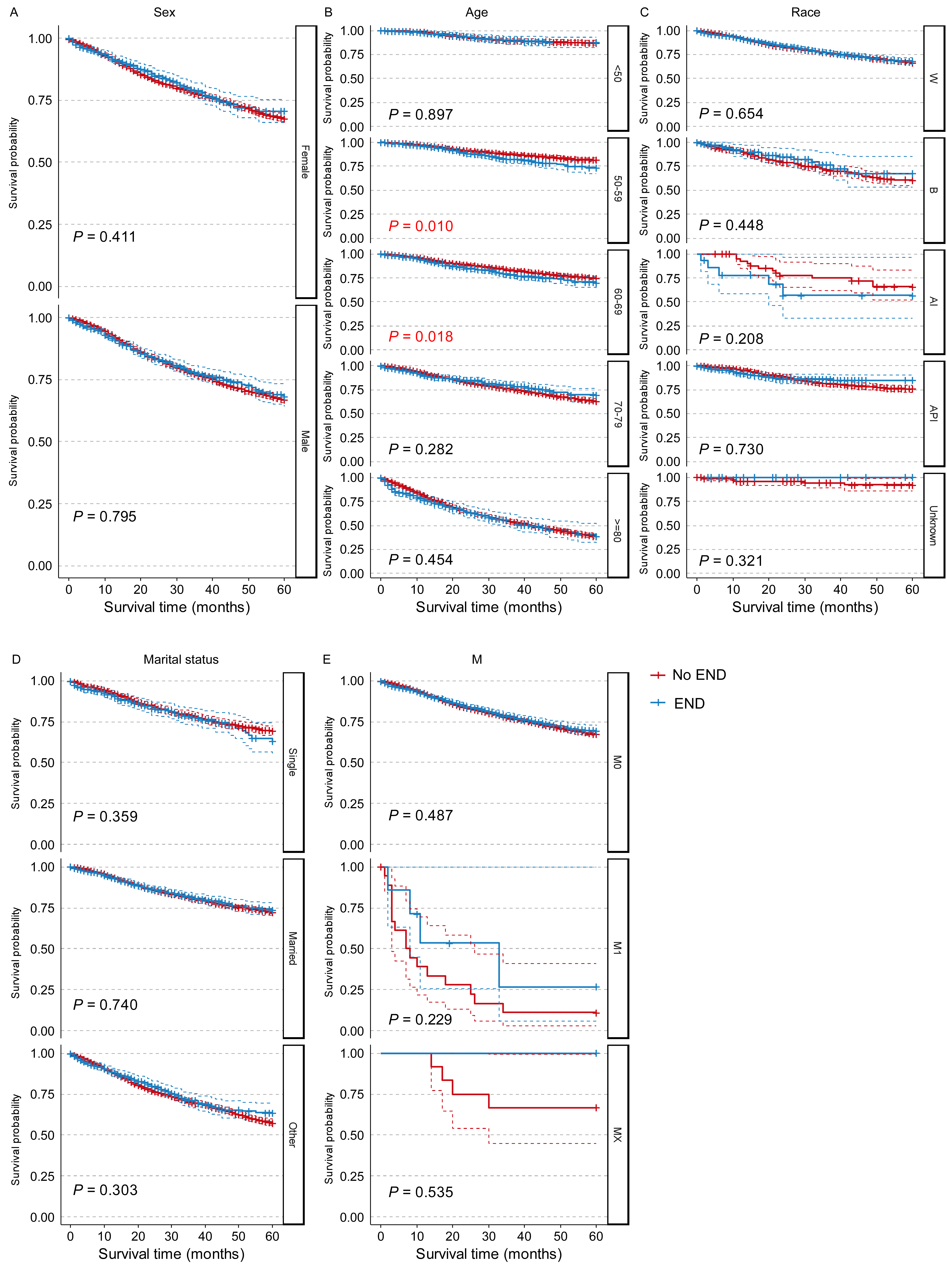


Supplementary Figure 9: OS of cN0 OSCC patients only performed primary sites surgery without radiation and chemotherapy according to (A) primary sites, (B) age, (C) race, (D) marital status and (E) M with END subgroups analysis.
